# Comparison of Ecological Momentary Assessment Versus Direct Measurement of E-Cigarette Use With a Bluetooth-Enabled E-Cigarette: A Pilot Study

**DOI:** 10.2196/resprot.6501

**Published:** 2017-05-29

**Authors:** Jennifer L Pearson, Hoda Elmasry, Babita Das, Sabrina L Smiley, Leslie F Rubin, Teresa DeAtley, Emily Harvey, Yitong Zhou, Raymond Niaura, David B Abrams

**Affiliations:** ^1^ Truth Initiative Schroeder Institute for Tobacco Research and Policy Studies Washington, DC United States; ^2^ Johns Hopkins Bloomberg School of Public Health Department of Health, Behavior, and Society Baltimore, MD United States; ^3^ Tobacco Center of Regulatory Science University of Maryland, School of Public Health College Park, MD United States; ^4^ American University Department of Psychology Washington, DC United States; ^5^ George Mason University Department of Anthropology Fairfax, VA United States; ^6^ Lombardi Comprehensive Cancer Center Georgetown University Washington, DC United States

**Keywords:** smoking, humans, tobacco products/utilization, electronic cigarettes, observational study, United States

## Abstract

**Background:**

Assessing the frequency and intensity of e-cigarette use presents special challenges beyond those posed by cigarette use. Accurate measurement of e-cigarette consumption, puff duration, and the stability of these measures over time will be informative for estimating the behavioral and health effects of e-cigarette use.

**Objective:**

The purpose of this pilot study was to compare the accuracy of self-reported e-cigarette puff counts collected via ecological momentary assessment (EMA) to objective puff count data collected by a Bluetooth-enabled e-cigarette device and to examine the feasibility and acceptability of using a second-generation e-cigarette among adult smokers.

**Methods:**

A total of 5 adult smokers were enrolled in a longitudinal parent study assessing how e-cigarette use affects cigarette use among e-cigarette–naïve smokers. Using a text message–based EMA system, participants reported e-cigarette puffs for 2 weeks. Participants were also given a Bluetooth-enabled e-cigarette (Smokio) that passively collected puff counts and puff duration. Comparisons between mean reports of Smokio (device-report) and EMA (self-report) use were evaluated using paired *t* tests. Correlation and agreement between device- and self-reports were evaluated using Pearson correlation and the concordance correlation coefficient (CCC), respectively. A linear mixed effect model was used to determine the fixed effect of timing and Smokio-reported daily puffs on report accuracy. We examined the relationship between time of day and reporting accuracy using Tukey's test for multiple pairwise comparisons.

**Results:**

A total of 5 African American participants, 4 men and 1 woman, who ranged in age from 24 to 59 years completed the study, resulting in 5180 observations (device-report) of e-cigarette use. At baseline, participants reported smoking for 5 to 25 years and consumed a mean of 7 to 13 cigarettes per day (CPD); 4 smoked within 30 minutes of waking. At the 30-day follow-up, CPD range decreased to 1 to 3 cigarettes; 4 participants reported past 7-day e-cigarette use, and 1 participant reported no cigarette smoking in the past 7 days. Over 2 weeks of e-cigarette use, participants took an average of 1074 e-cigarette (SD 779.0) puffs per person as captured by the device reports. Each participant took a mean of 75.0 (SD 58.8) puffs per day, with each puff lasting an average of 3.6 (SD 2.4) seconds. Device reports captured an average of 33.3 (SD 47.8) more puffs per person per day than the self-reported e-cigarette puffs. In 87% of days, participants underestimated the number of puffs they had taken on the Smokio. There was significant moderate correlation (*r*=.47, *P*<.001) but poor agreement (*p*_c_=0.31, 95% CI 0.15-0.46) between the device- and self-reported data. Reporting accuracy was affected by amount and timing of e-cigarette use.

**Conclusions:**

Compared to self-reported e-cigarette use, the Bluetooth-enabled device captured significantly more e-cigarette use and allowed for examination of puff duration in addition to puff counts. A Bluetooth-enabled e-cigarette is a powerful and feasible tool for objective collection of e-cigarette use behavior in the real world.

## Introduction

There has been a rapid increase in lifetime and past 30-day adult use of e-cigarettes in the United States since their introduction in 2007 [1-3]. The highest prevalence of e-cigarette use is among current and former cigarette smokers [4-6], who most commonly report use to reduce cigarette consumption, quit smoking, or prevent smoking relapse [6-10].

Much of the scientific literature on e-cigarette use stems from national surveys and laboratory studies, which are limited by recall bias or are not generalizable to e-cigarette use behavior in the real world. Ecological momentary assessment (EMA) is an intensive longitudinal method that samples participant behaviors and experiences to reveal how individual differences and within-person processes interact to produce a behavioral outcome such as quitting smoking [11]. EMA reduces recall bias and threats to generalizability common to retrospective surveys and laboratory studies by sampling participant behavior and experience in the time and place where the behavior or experience occurs. In a comparison of methods to assess daily cigarette use, EMA best correlates with biomarkers of cigarette smoking, suggesting that this is a valid method to assess cigarette smoking frequency [12]. EMA has been extensively applied to tobacco control and smoking cessation treatment research to better understand phenomena such as smoking patterns, the smoking cessation process, and the interaction between the built environment and smoking craving [12,13].

Accurate measurement of e-cigarette consumption, puff duration, and the stability of these measures over time will be informative for estimating the behavioral and health effects of e-cigarette use. Because EMA has been applied in cigarette smoking research, EMA could also be a powerful tool to understand e-cigarette use in isolation and in combination with other tobacco products (dual use). To date, no studies have employed EMA assessment of e-cigarette use in analyses, and only one study has used daily reports to examine within-person variation in e-cigarette use among smokers; in that study, only the presence or absence of e-cigarette use in that day was assessed, without attention to frequency or intensity of use [14]. A more fine-grained assessment of e-cigarette use in its physiological, social, and environmental context will yield a better understanding of the individual differences and e-cigarette product features that promote or discourage use and will be informative of any future US Food and Drug Administration Center for Tobacco Products (FDA CTP) e-cigarette regulation.

Measurement of e-cigarette use poses challenges beyond those posed by measuring cigarette smoking. The term e-cigarette encompasses an array of products with different performance characteristics. Unlike cigarettes, which have a distinct beginning and end point, an e-cigarette could last several days before it needs to be refilled or discarded. Asking about the number of puffs in an e-cigarette use session may be an adequate measure of e-cigarette use intensity; however, it is unknown whether adult smokers can reliably report e-cigarette puff counts. The purpose of this pilot study was to compare the accuracy of self-reported e-cigarette puff counts collected via EMA to objective puff count data collected by a Bluetooth-enabled e-cigarette device and to examine the feasibility and acceptability of using a second-generation e-cigarette among adult smokers.

## Methods

### Study Design

Data from this study come from a pilot embedded in a longitudinal study (Mixed Method E-Cigarette [Moment] Study). Details on the study protocol and procedures are available elsewhere [15]. Briefly, the Moment Study was a 6-week intensive longitudinal study that employed a mixed methods design to yield an in-depth description of the e-cigarette initiation process among adult smokers. Participants completed 4 in-person visits, followed by an online follow-up survey at 30 days after the final in-person visit. Participants in the Moment Study were provided with NJOY King disposable e-cigarettes at the second in-person visit and self-reported their subsequent e-cigarette and cigarette consumption via text message EMA. For this pilot study, 5 participants were provided with a Bluetooth-enabled e-cigarette tank system (Smokio brand) that passively recorded puff count and puff duration data. Like participants in the parent study, pilot study participants also submitted self-reported e-cigarette and cigarette consumption data via text message EMA. The Moment Study’s mixed method design featured concurrent collection of multiple data streams, including (1) EMA, (2) geotracking, (3) in-depth interviews, and (4) biosamples. Geotracking and biosample data are not reported in this study and will not be discussed further.

### Study Population and Recruitment

Eligible individuals were English-speaking adults aged 18 years or older residing in the Washington, DC, metro area who smoked at least 8 cigarettes a day for the past 5 years. To simplify EMA tobacco use reports, we excluded polytobacco users, defined as having smoked a little cigar/cigarillo, large cigar, or hookah more than 5 times in the last 30 days or used smokeless tobacco in the past 30 days. Additional eligibility criteria included (1) no e-cigarette use in the last 30 days, (2) interest in trying an e-cigarette, and (3) report interest in quitting cigarette smoking in the next 30 days at the initial screening. To facilitate EMA data collection, participants were required to use a cell phone daily and have an unlimited text message plan. Participants were recruited via public online postings, paid advertisements, and physical flyers. Recruitment documents directed potential participants to an online screening survey (www.ecigstudy.org).

### Procedures

**Figure 1:**
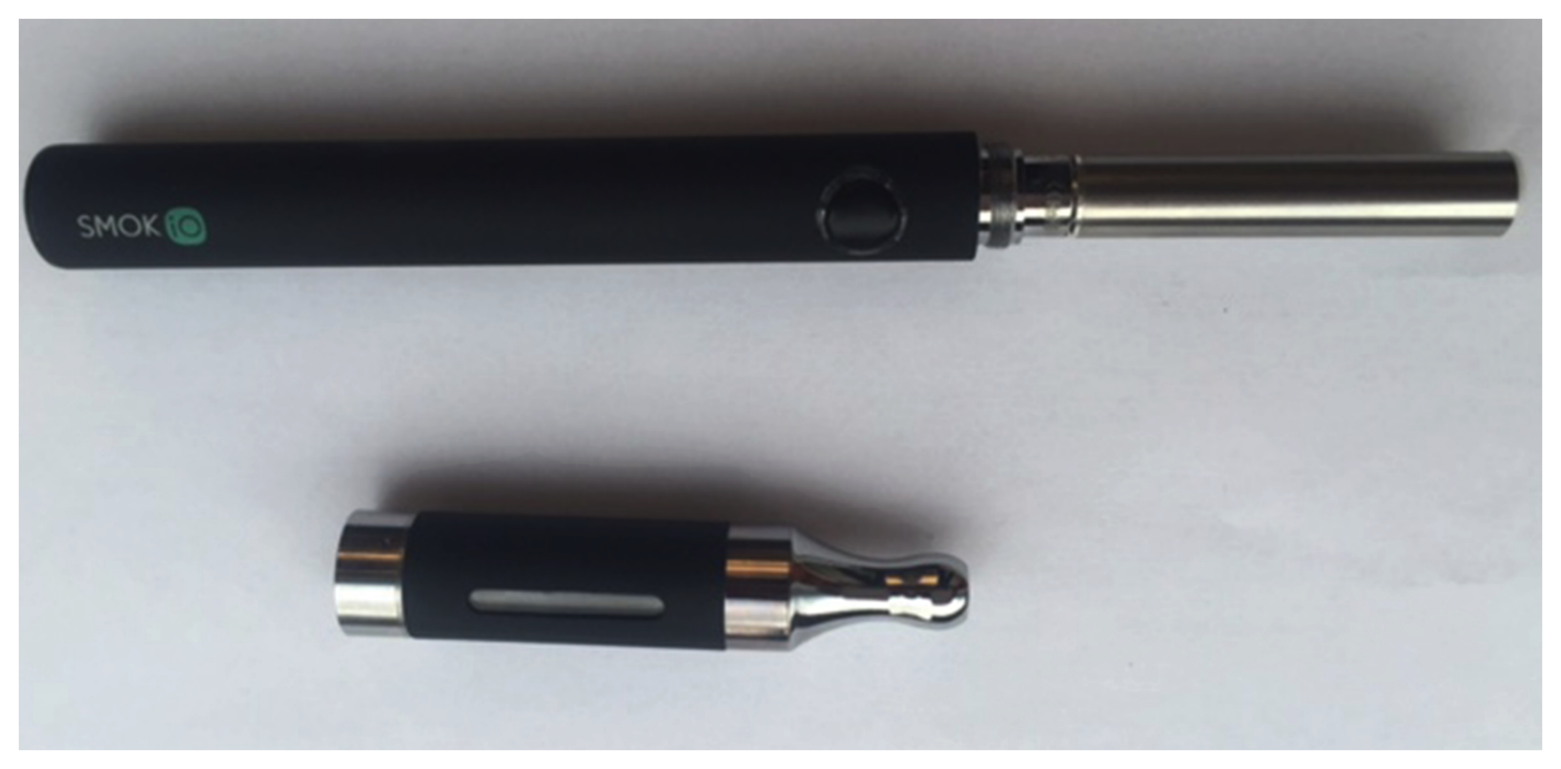
The Smokio battery/cartomizer combination (top) was given to participants during the study; the Smokio tank (bottom) was provided to participants at the end of the study.

In-person procedures consisted of 4 office visits. During the baseline visit, participants completed the informed consent process and confirmed current smoking status with an exhaled carbon monoxide test. A research assistant (RA) registered participant phones to receive EMA text messages and trained participants on how to respond to the EMA random texts and self-initiated tobacco use reports. At the second office visit, participants were provided with 2 Smokio batteries (Figure 1) and 10 prefilled cartomizers (single coil, 1.5 ohm, 510-threaded, Smok brand) of 1.8% nicotine fluid in tobacco (AVAIL VA Pure) or menthol flavor (AVAIL Port Royal), depending on their cigarette flavor preference. The RA trained participants on how to use the Smokio and asked participants to take a minimum of 3 puffs a day for the next week. Prefilled cartomizers were provided to participants rather than the Smokio tank and 10 mL of nicotine fluid to simplify use of the device. Nicotine fluid was purchased from AVAIL Vapor (www.availvapor.com) in a 70%/30% propylene glycol/vegetable glycerin mix and was independently verified by an analytic chemist at Virginia Commonwealth University as containing an average of 17.1 mg/mL of nicotine (6 vials with a range of nicotine concentrations between 16.8 and 17.7 mg/mL). At the third office visit, participants received an additional 10 prefilled cartomizers and instructions to use the device as desired. At the last office visit, participants were provided with an empty Smokio tank (Figure 1) but no additional nicotine fluid or cartridges. At 30 days after their last contact with the study, participants were sent a reminder email with an embedded Web link to take the online follow-up survey. All study procedures were reviewed and approved by Chesapeake Institutional Review Board (Pro00008526).

### Measurement Instruments

This study employed 2 types of active EMA data collection: (1) participant-initiated cigarette and e-cigarette use reports and (2) system-initiated random prompts to assess mood and craving. Random prompt data are not reported in this manuscript; more information on these methods is available elsewhere [15]. Participant-initiated reports collected information on cigarette consumption (weeks 1-3), e-cigarette consumption (weeks 2-3), satisfaction derived from the reported product (weeks 1-3), and desire to use the opposite product (eg, desire to smoke after using the e-cigarette; weeks 2-3). Participants were instructed to self-initiate an e-cigarette puff report when they “put down the e-cigarette and did not intend to pick it up again in a while.” Participants texted #cig (to report cigarette use in week 1) or #both (to report cigarette or e-cigarette use in weeks 2-3) to the study system phone number, which initiated a short series of questions about the recent cigarette or e-cigarette use. Questions included “About how many drags did you take on the e-cig?” and “How many minutes ago did you finish using the e-cig?” Participants could initiate an unlimited number of reports in a day and could report cigarette or e-cigarette use at any time. As participants had to smoke at least 8 cigarettes per day to be eligible for the study, they were encouraged to make an average of 6 cigarette or e-cigarette reports per day and incentivized with an additional $10 per week if they self-initiated at least 42 reports each week.

Both streams of data were collected via text messages on participant personal cell phones. The EMA data collection system returned an error message to prevent participants from skipping items or entering out-of-range values. All EMA entries were stamped with the time, date, and geolocation of the report and were uploaded to Truth Initiative’s secure server via an encrypted representational state transfer application program interface.

Smokio (Figure 1) is a Bluetooth-enabled e-cigarette that records puff counts (ignition button clicks) and puff duration (span of time in milliseconds that the ignition button is depressed). Devices were paired with the Smokio app on participant cell phones, which pushed data from the Smokio to the Cloud. If the paired cell phone was not within range of the Smokio, the device cached data until the phone was nearby. Participants indicated that Smokio could share their puff data with the study by entering the study’s email address into the Smokio app. After obtaining permission, the RA downloaded a .csv file of participant puff data from Smokio’s Web portal. Participants removed the study’s permission to access their puff data at the last study visit.

An RA conducted in-depth interviews with all participants at week 1, 2, and 3 office visits. Among other topics, the RA asked participants about their sensory and social experience using the e-cigarette and any difficulties they had using the device, such as remembering to keep it charged. Interviews did not exceed 30 minutes. Both the baseline and follow-up surveys were computer-assisted self-interview surveys. Survey questions assessed sociodemographics, tobacco and e-cigarette use history, tobacco and e-cigarette use beliefs and cognitions, tobacco product and e-cigarette harm perceptions, alcohol use, and health status.

### Analyses

Descriptive statistics were used to characterize the sample in terms of demographic characteristics (sex, age, race, and education), baseline factors (years smoked, cigarettes per day, nicotine dependence, and other tobacco use) and follow-up smoking/e-cigarette use (cigarettes per day, e-cigarette use, and point prevalence abstinence). All EMA e-cigarette puff reports (self-reports and device-reports) were aggregated at the day level. Comparisons between day-level mean reports of e-cigarette device-recorded puffs and self-reported puffs were evaluated using paired *t* tests. Correlation and agreement between e-cigarette device reports and self-reported puff counts were evaluated using Pearson correlation and the concordance correlation coefficient (CCC), respectively. The CCC is a statistic to assess interrater reliability and was used to assess agreement between the device-reported and self-reported puff counts at the day level. The CCC ranges from –1 (complete negative agreement) to 1 (complete agreement), with 0 indicating no agreement.

After checking the descriptive statistics, we examined the extent to which timing and device-reported daily puffs influenced self-report accuracy. A linear mixed effect model was used to determine the fixed effect of timing and Smokio-reported daily puffs on report accuracy by taking the random effect of each individual into consideration. To find out the best and worst timing under each time window cases, Tukey's test was used for multiple pairwise comparisons. All statistical analyses were performed in R (The R Foundation) and SAS version 9.4 (SAS Institute Inc); figures were created in R, SAS 9.4 and JMP version 10.0.2 (SAS Institute Inc). Statistical significance was set to a *P* value of .05.

## Results

### Participant Characteristics

A total of 5 African American participants, 4 men and 1 woman, with an age range of 24 to 59 years completed this pilot study (Table 1). At baseline, participants reported having smoked for 5 to 25 years and consumed between 7 and 13 cigarettes per day; 4 out of 5 participants smoked within 30 minutes of waking. All participants used his or her Smokio throughout the 2-week e-cigarette observation period, and no one lost a device. At the 30-day follow-up, cigarettes per day (CPD) range decreased to 1 to 3 cigarettes, with 4 participants reporting any past 7-day e-cigarette use and 1 participant reporting no past 7-day cigarette smoking.

### E-Cigarette Puff Data

Over 2 weeks of e-cigarette use, the Smokio device captured 5180 e-cigarette puff observations. Participants took an average of 1074 (SD 779.0) e-cigarette puffs per person, with a mean of 75.0 (SD 58.8) puffs per day and 536 (SD 377.9) puffs per week, with each puff lasting an average of 3.6 (SD 2.4) seconds (device data). The average number of device- and self-reported puffs per person per day did not vary by week.

Table 2 presents average e-cigarette puffs per day, comparing the 2 data collection methods. Smokio captured an average of 33.3 (SD 47.8) more puffs per person per day than the self-reported e-cigarette puffs. Smokio identified significantly more daily puffs per person than self-reports overall (*P*<.001), at week 2 (*P*<.001), and at week 3 (*P*<.001). In 87% of daily reports, participants underestimated the number of puffs they had taken on the Smokio. Across individuals and days, there was a significant moderate correlation (*r*=.47, *P*<.001) between the device-reported and self-reported puff count data; however, there was poor agreement (*p*_c_=0.31, 95% CI 0.15-0.46) between device-reported and self-reported puff counts. Given the variability in puff counts by participant, CCC was further assessed at the individual level; the CCC ranged from virtually no agreement (Participant 2; *p*_c_=0.001, 95% CI –0.10 to 0.11) to high agreement (Participant 3; *p*_c_=0.91, 95% CI 0.76-0.97) between the device-reported and self-reported puff counts.

**Table 1:** Participant characteristics at baseline and 30-day follow-up (n=5).

Characteristics			Number
**Sociodemographics**			
	Female, n		1
	Age, range		24-59
	African American, n		5
	**Education, n**		
		High school or less	1
		Some college or more	4
**Tobacco use (baseline)**			
	Years smoked, range		5-25
	CPD^a^, range		7-13
	Smoke within 30 minutes of waking, n		4
**Tobacco use (follow-up)**			
	CPD, range		1-3
	Past 7-day e-cigarette use, n		4
	7-day cigarette smoking PPA^b^, n		1
	**Change in cigarette use, n**		
		Increase	0
		No change	1
		Decrease	4
	**Change in e-cigarette use, n**		
		Increase	1
		No change	0
		Decrease	4

^a^CPD: cigarettes per day.

^b^PPA: point prevalence abstinence.

**Table 2:** Descriptive statistics of e-cigarette use by week of study, comparing e-cigarette use captured by the Smokio device to self-reported e-cigarette use.

Puffs per person per day	Device-reported	Self-reported	% difference	*P* value^a^
Overall, mean (SD)	75.0 (58.8)	48.0 (32.6)	56.25	<.001
Week 2, mean (SD)	69.9 (53.1)	47.3 (31.0)	47.78	<.001
Week 3, mean (SD)	80.1 (64.1)	48.7 (34.6)	64.48	<.001

^a^Paired *t* test results.

**Figure 2:**
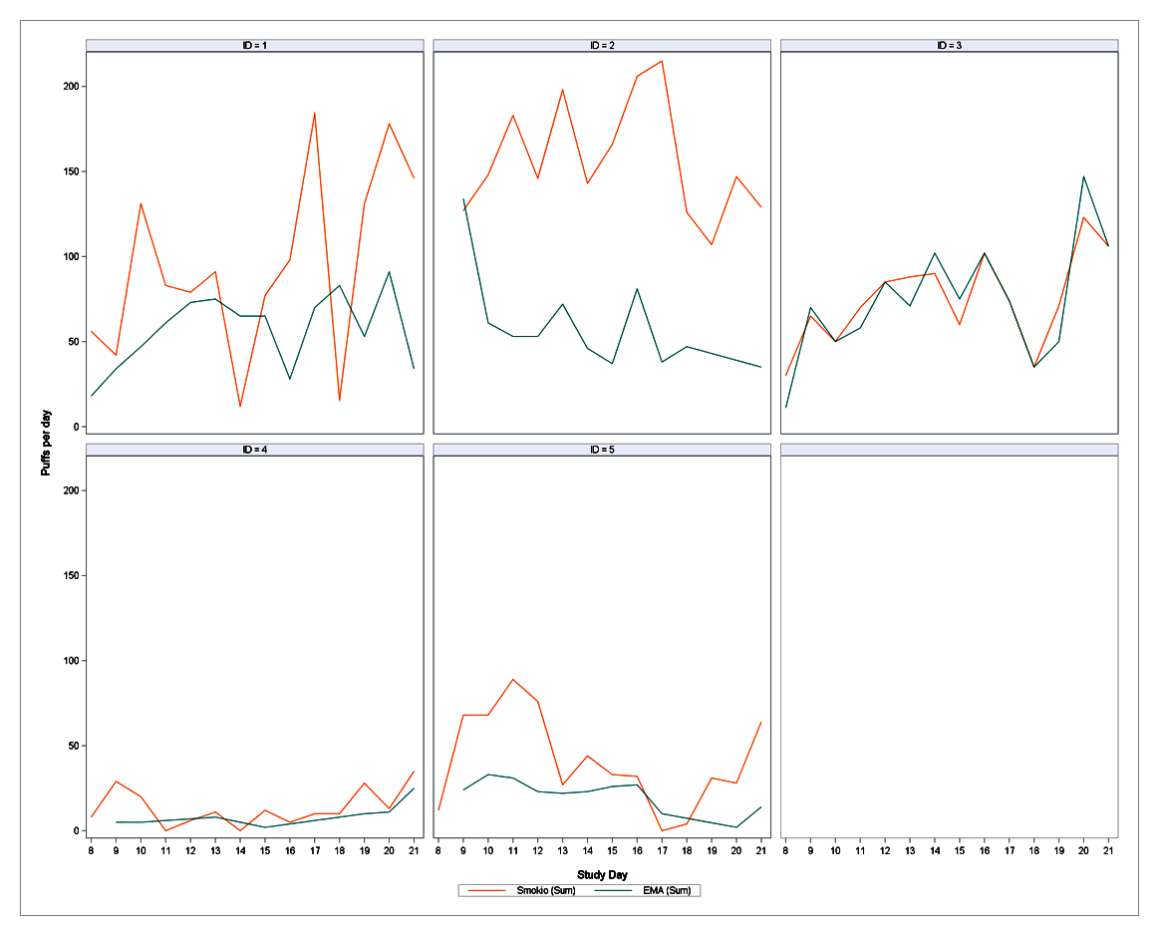
Panel plot of e-cigarette puffs per day by participant and study day comparing device-reported puffs (orange line) to self-reported puffs (blue line).

Figure 2 presents number of e-cigarette puffs per day by participant, comparing device-reported puffs to self-reported puffs. These plots reveal significant variability in within- and between-person daily e-cigarette consumption and highlight participants’ tendency to underreport e-cigarette puffs. For example, Participant 2 consumed the greatest number of puffs but did not accurately report his puffs via EMA; in contrast, Participant 4 was a light Smokio user and his EMA puff reports closely followed his Smokio-recorded puffs. Participant 3, a moderate Smokio user, was remarkably accurate in her e-cigarette puffs. In an examination of prediction accuracy by the number of Smokio-reported daily puffs, the percentage difference between Smokio and self-reported e-cigarette puffs increased by 4.5% for every 1-puff increase captured by the Smokio (*P*<.001). Comparing reporting accuracy by time of day (day divided into 12 2-hour increments), participants’ self-reports were most accurate from 4 PM to 6 PM and least accurate from 6 AM to 8 AM. In this small sample, reporting accuracy depended on the amount and timing of e-cigarette use.

### Device Acceptability

All 5 of the participants felt the device was convenient and acceptable, with none reporting problems with turning the device off/on or keeping it charged. Participants were struck by their ability to use the Smokio in places where they could not smoke a cigarette.

I kind of like the fact that I can kind of stay in the crowd and still get my nicotine without offending other people.

Participant 2

I could smoke it like absolutely anywhere…it didn’t bother anybody.

Participant 5

Participants also positively described the taste of the AVAIL e-liquid.

It tasted menthol-y enough to where it satisfied the nicotine taste, my body's craving.

Participant 2

It was a pretty good experience for first time using. [It gets]…that sweet menthol taste out of it. So it’s pretty good actually.

Participant 3

Participant 1 stated that it “was weird trying to get used to the taste of it.” While Participant 2 reported “holding it was kind of odd because it wasn't the size of a cigarette,” no one disliked the Smokio because of its size or weight. All participants felt that using the device suppressed their urge for a cigarette.

When I did smoke the Smokio, it took away a lot of cravings and urges, made me feel better.

Participant 1

It satisfies my craving when I need it.

Participant 4

## Discussion

### Principal Findings

Compared to self-initiated e-cigarette puff reports, the Smokio proved to be a far superior method to collect e-cigarette puff data. However, the moderate correlation (*r*=.47, *P*<.001) and high agreement between device-reported and self-reported puff data for some participants (eg, Participant 3; *p*_c_=0.91, 95% CI 0.76-0.97) demonstrate that self-reported e-cigarette puff data may be a feasible method for collecting naturalistic e-cigarette use data, especially among low-level users. Self-initiated puff reports may not be an optimal data collection method for high-level e-cigarette users. However, the exceptional agreement between Participant 3’s Smokio-captured and self-initiated reports suggests that research participants may be able to improve their self-report precision with training. Future investigation of how to improve the validity of self-reported puff counts would be of great utility to the field. It may be that other approaches to understanding naturalistic use, such as an EMA coverage approach, collection of used cartomizers, or assessment of weekly or usual consumption may outperform the self-initiated puff report used in this study.

In comparing device-reported to self-reported data, other consistent trends were revealed. First, we observed that participants underestimated the number of e-cigarette puffs consumed in nearly 90% of study days. While missing data is never preferred, a consistent pattern to missing data can be accounted for in the analyses and interpretation of results. Second, despite a burdensome design, participants did not reduce their average self-reported e-cigarette puff counts between weeks, indicating that there was minimal fatigue in EMA e-cigarette reporting. While Smokio captured more e-cigarette puff data than self-reports, the between-person average difference between the two methods remained steady between the second and third weeks of data collection. If objective measurement of e-cigarette use using a device like Smokio is cost prohibitive or otherwise not feasible (eg, participants are provided with “cigalike” e-cigarettes, or e-cigarettes with small batteries that are often disposable), self-reported e-cigarette puffs may be an acceptable alternative as long as the research question and design allow for underreporting of e-cigarette use and variation in the accuracy of reporting between individuals by heaviness of e-cigarette use.

Data from in-depth interviews with our 5 pilot participants demonstrate that the Smokio device and e-liquid choices were acceptable and convenient. Participants reported liking the taste and experience of using the Smokio, although one individual initially found using the device awkward. Participants also commented on the Smokio’s ability to alleviate craving and the appeal of feeling free to use the device in places where they could not smoke cigarettes. None of our participants reported trouble with keeping the device charged nor did they find operation of the device challenging. Continued use of an e-cigarette at follow-up also suggests that the device was acceptable, although we cannot be certain that participants continued to use the Smokio from the study or some other e-cigarette device. At the 30-day follow-up, it is notable that all participants reported that they smoked fewer cigarettes than when they enrolled in the study, with the average CPD dropping from 9 to 1.8.

### Limitations

This pilot study has several limitations. First, as a pilot study, conclusions are based on only 5 individuals. Results are intended to inform measurement of e-cigarette use in EMA studies and should not be interpreted beyond this purpose. Second, we assumed that the device-recorded puffs were the gold standard method for assessing naturalistic e-cigarette use; however, we did not conduct a formal laboratory assessment of the Smokio puff counter or puff duration measurement and thus cannot be certain of the precision of the device. Additionally, as of summer 2016, the Smokio (now called Vap.io) is no longer available for purchase in the United States. Other devices, such as several Joytech products, collect puff count data but do not currently sync data and push to a remote server, which allows real-time monitoring of e-cigarette use behavior and reduces data loss if a participant misses an in-person visit. Future collaboration with private companies or independent developers will be necessary to create a product with capabilities similar to the Smokio. Research funders should consider supporting the development of a range of e-cigarette device types that passively capture user data, including puff counts and puff duration. We also note that Participant 3 was remarkably accurate in her self-reports. We did not collect information that explains why her self-reports outperformed those of the other participants; however, participants did know that the Smokio counted their puffs—that awareness could have encouraged counting. It is also possible that novel users are more aware of their puff patterns than more established users; thus, these conclusions may not transfer to established e-cigarette users. We also did not collect information on the amount of e-liquid consumed by each participant, which would be an additional helpful source of data to compare against the Smokio-reported puffs. Finally, our instruction to participants to initiate an e-cigarette self-report when they “put down the e-cigarette and did not intend to pick it up again in a while” may not translate to established vapers who “graze” on their devices throughout the day. In this population, an EMA coverage approach or, ideally, provision of a Smokio-like device would be the best option to capture puffing data.

### Conclusions

Compared to self-reported e-cigarette use, the Bluetooth-enabled device captured significantly more e-cigarette use and allowed for examination of puff duration in addition to puff counts. A Bluetooth-enabled e-cigarette is a powerful and feasible tool for objective collection of e-cigarette use behavior in the real world. As e-cigarette users adopt more sophisticated devices, researchers should consider harnessing the existing capabilities of these devices to aid data collection.

## Abbreviations

CCC: concordance correlation coefficient
CPD: cigarettes per day
CTP: Center for Tobacco Products
EMA: ecological momentary assessment
FDA: Food and Drug Administration
RA: research assistant
